# Supplementary material for: Eye-brain connections revealed by multimodal retinal and brain imaging genetics
Source: Nat Commun. 2024 Jul 18;15:6064. doi: 10.1038/s41467-024-50309-w (PMC11258354; doi:10.1038/s41467-024-50309-w)
Supplement: Supplementary file 3 — Description of Additional Supplementary Files [file 41467_2024_50309_MOESM3_ESM.pdf]

Description of Additional Supplementary Files

File Name: Supplementary Data 1
Description: ID of brain and retinal imaging traits used in phenotypic association analysis.

File Name: Supplementary Data 2
Description: Significant eye-brain phenotypic associations between retinal imaging traits and brain MRI traits.

File Name: Supplementary Data 3
Description: SNP-based heritability of 156 retinal imaging traits.

File Name: Supplementary Data 4
Description: Independent (LD < 0.1) significant associations for retinal imaging traits.

File Name: Supplementary Data 5
Description: Validating significant retinal imaging associations in independent validation GWAS datasets.

File Name: Supplementary Data 6
Description: Performance of polygenic risk scores of the 156 retinal imaging traits.

File Name: Supplementary Data 7
Description: Genetic overlaps of eye-brain connections.

File Name: Supplementary Data 8
Description: Summary of the shared genetic effects between retinal imaging traits and brain phenotypes.

File Name: Supplementary Data 9
Description: Genetic correlations between retinal imaging traits and brain-related complex traits and diseases analyzed in previous studies.

File Name: Supplementary Data 10
Description: LDSC partitioned heritability enrichment analysis.

File Name: Supplementary Data 11
Description: Mendelian randomization analysis between retinal imaging traits and brain diseases.

File Name: Supplementary Data 12
Description: Predicting complex traits and diseases using retinal imaging traits.
