# Supplementary material for: Eye-brain connections revealed by multimodal retinal and brain imaging genetics
Source: Nat Commun. 2024 Jul 18;15:6064. doi: 10.1038/s41467-024-50309-w (PMC11258354; doi:10.1038/s41467-024-50309-w)
Supplement: Supplementary file 16 — Reporting Summary [file 41467_2024_50309_MOESM16_ESM.pdf]

nature portfolio

Corresponding author(s): Bingxin Zhao

Last updated by author(s): May 20, 2024

Reporting Summary

Nature Portfolio wishes to improve the reproducibility of the work that we publish. This form provides structure for consistency and transparency in reporting. For further information on Nature Portfolio policies, see our Editorial Policies and the Editorial Policy Checklist.

Statistics

For all statistical analyses, confirm that the following items are present in the figure legend, table legend, main text, or Methods section.

n/a	Confirmed	
☐	☒	The exact sample size (*n*) for each experimental group/condition, given as a discrete number and unit of measurement
☐	☒	A statement on whether measurements were taken from distinct samples or whether the same sample was measured repeatedly
☐	☒	The statistical test(s) used AND whether they are one- or two-sided<br>*Only common tests should be described solely by name; describe more complex techniques in the Methods section.*
☐	☒	A description of all covariates tested
☐	☒	A description of any assumptions or corrections, such as tests of normality and adjustment for multiple comparisons
☐	☒	A full description of the statistical parameters including central tendency (e.g. means) or other basic estimates (e.g. regression coefficient) AND variation (e.g. standard deviation) or associated estimates of uncertainty (e.g. confidence intervals)
☐	☒	For null hypothesis testing, the test statistic (e.g. *F*, *t*, *r*) with confidence intervals, effect sizes, degrees of freedom and *P* value noted<br>*Give P values as exact values whenever suitable.*
☐	☒	For Bayesian analysis, information on the choice of priors and Markov chain Monte Carlo settings
☐	☒	For hierarchical and complex designs, identification of the appropriate level for tests and full reporting of outcomes
☐	☒	Estimates of effect sizes (e.g. Cohen's *d*, Pearson's *r*), indicating how they were calculated

Our web collection on statistics for biologists contains articles on many of the points above.

Software and code

Policy information about availability of computer code

Data collection

There is no clear distinction between software/code used for data collection vs data analysis, thus we list all software here:
UKB Brain Imaging Pipeline, https://git.fmrib.ox.ac.uk/falmagro/UK_biobank_pipeline_v_1;
UKB Eye Imaging Pipeline, https://biobank.ndph.ox.ac.uk/showcase/label.cgi?id=100016;
UK Biobank Brain Imaging - Online Resources, https://www.fmrib.ox.ac.uk/ukbiobank;
Transfer learning models for retinal fundus images: https://github.com/mkirchler/transferGWAS;
GCTA and fastGWAS (1.94.0beta), https://yanglab.westlake.edu.cn/software/gcta/;
PLINK (1.90 beta), https://www.cog-genomics.org/plink/;
FUMA GWAS (version v1.3.8), https://fuma.ctglab.nl/;
LDSC (v1.0.1), https://github.com/bulik/ldsc/;
LAVA (v0.1.0), https://github.com/josefin-werme/LAVA;
NHGRI-EBI GWAS Catalog, https://www.ebi.ac.uk/gwas/home;
MetaBrain, https://www.metabrain.nl/;
TwoSampleMR: https://mrcieu.github.io/TwoSampleMR/;
IEU GWAS database: https://gwas.mrcieu.ac.uk/;
coloc (version 5): https://github.com/chr1swallace/coloc

Data analysis

Please see above.

For manuscripts utilizing custom algorithms or software that are central to the research but not yet described in published literature, software must be made available to editors and reviewers. We strongly encourage code deposition in a community repository (e.g. GitHub). See the Nature Portfolio guidelines for submitting code & software for further information.

Data

Policy information about availability of data

All manuscripts must include a data availability statement. This statement should provide the following information, where applicable:

- Accession codes, unique identifiers, or web links for publicly available datasets
- A description of any restrictions on data availability
- For clinical datasets or third party data, please ensure that the statement adheres to our policy

The GWAS summary statistics of retinal imaging traits generated in this study have been deposited in the Zenodo database under accession code 11217687 (https://doi.org/10.5281/zenodo.11217687). The GWAS summary statistics of brain MRI traits can be freely downloaded at BIG-KP (https://bigkp.org/). The individual-level UK Biobank imaging data used in this study can be obtained from https://www.ukbiobank.ac.uk/.

Research involving human participants, their data, or biological material

Policy information about studies with human participants or human data. See also policy information about sex, gender (identity/presentation), and sexual orientation and race, ethnicity and racism.

Reporting on sex and gender	Both male and female were used in the study. This was considered in the study design and was determined based on self-report. We performed sex-specific analysis in our project.
Reporting on race, ethnicity, or other socially relevant groupings	The UKB database has a variable ("Data-Field 21000" https://biobank.ndph.ox.ac.uk/showcase/field.cgi?id=21000) for ethnic background and its accuracy has been verified using genotyping data in Bycroft et al. (Nature, 2018, PMID: 30305743) and was used widely in previous studies. We used this variable to perform ancestry-specific GWAS analysis.
Population characteristics	Approximately half a million white British ancestry from the UKB study who aged between 40 and 69 between 2006 to 2010.
Recruitment	Recruitment details and dataset overviews can be found in https://doi.org/10.1371/journal.pmed.1001779.
Ethics oversight	The wide consultation, rigorous Ethics and Governance Framework, and Ethics and Governance Council oversight role have been essential in paving the way for UK Biobank to accomplish obtaining the multiple ethical and regulatory approvals required for participant recruitment, sample and data storage, linkages to routine health care data, enhancement studies, and the provision of access to data and samples for approved researchers. Substantial amounts of time, resources, patience, tenacity, and evidence of feasibility and/or acceptability from smaller scale pilot studies have also been required to provide regulatory bodies with the reassurance that they need of UK Biobank's rigorous approach and commitment to protecting the interests of its participants within an acceptable legal and ethical framework (details can be found in https://doi.org/10.1371/journal.pmed.1001779). Informed consent was obtained by participants.

Note that full information on the approval of the study protocol must also be provided in the manuscript.

Field-specific reporting

Please select the one below that is the best fit for your research. If you are not sure, read the appropriate sections before making your selection.

☒ Life sciences ☐ Behavioural & social sciences ☐ Ecological, evolutionary & environmental sciences

For a reference copy of the document with all sections, see nature.com/documents/nr-reporting-summary-flat.pdf

Life sciences study design

All studies must disclose on these points even when the disclosure is negative.

Sample size	No power calculation was needed in advance. We used all samples passing standard quality controls (please see below). We have performed multiple testing adjustments whenever appropriate to ensure false discovery rates were controlled.
Data exclusions	Full details of data exclusions can be found in the Methods section. For brain imaging phenotype data and GWAS data, all data exclusions have been performed in previous studies. Details can be found in Zhao et al. (https://doi.org/10.1038/s41588-019-0516-6), Zhao et al. (https://doi.org/10.1126/science.abf3736), Zhao et al. (https://doi.org/10.1101/2021.07.27.21261187), Zhao et al. (https://doi.org/10.1101/2021.11.01.21265779). As suggested, we used the data in Data-Fields 28552 & 28553 to perform quality control for OCT measures by keeping images with an image quality score > 45. We further only keep the OCT measures with a sample size > 30,000. In all the OCT measures and fundus image traits, the values greater than five times the median absolute deviation from the median were treated as outliers and removed.
Replication	Phenotypic association analyses results have been replicated using a hold-out independent dataset. For GWAS of eye imaging traits, we performed validations using 1) the UKB European but non-British subjects (average n = 5,320) and 2) UKB non-European subjects (average n = 6,490). Reproducibility of brain imaging traits and GWAS results of imaging traits have been examined in previous studies. Details can be found in Zhao et al. (https://doi.org/10.1038/s41588-019-0516-6), Zhao et al. (https://doi.org/10.1126/science.abf3736), Zhao et al. (https://doi.org/10.1101/2021.07.27.21261187), Zhao et al. (https://doi.org/10.1101/2021.11.01.21265779).

Randomization	All the datasets are from observational studies, and we used all samples available after data exclusions listed above. Therefore, there is no equivalent process of randomization in the present analysis.
Blinding	The data are from observational studies and not from controlled randomized studies, thus there is no step equivalent to blinding involved.

Reporting for specific materials, systems and methods

We require information from authors about some types of materials, experimental systems and methods used in many studies. Here, indicate whether each material, system or method listed is relevant to your study. If you are not sure if a list item applies to your research, read the appropriate section before selecting a response.

Materials & experimental systems

n/a	Involved in the study
☒	☐ Antibodies
☒	☐ Eukaryotic cell lines
☒	☐ Palaeontology and archaeology
☒	☐ Animals and other organisms
☒	☐ Clinical data
☒	☐ Dual use research of concern
☒	☐ Plants

Methods

n/a	Involved in the study
☒	☐ ChIP-seq
☒	☐ Flow cytometry
☐	☒ MRI-based neuroimaging

Plants

Seed stocks	NA
Novel plant genotypes	NA
Authentication	NA

Magnetic resonance imaging

Experimental design

Design type	This study made use of imaging data from brain magnetic resonance imaging.
Design specifications	Details can be found in Alfaro-Almagro et al. (https://doi.org/10.1016/j.neuroimage.2017.10.034).
Behavioral performance measures	Behavioral performance measures were not used in this study.

Acquisition

Imaging type(s)	Brain structural MRI, brain diffusion MRI, and brain functional MRI
Field strength	3T
Sequence & imaging parameters	Details can be found in Miller et al. (doi:10.1038/nn.4393) and Alfaro-Almagro et al. (https://doi.org/10.1016/j.neuroimage.2017.10.034).
Area of acquisition	The whole heart and brain scans were used.

Diffusion MRI ☒ Used ☐ Not used

Parameters	For each of the two diffusion-weighted shells, 50 distinct diffusion-encoding directions were acquired, two b-values (b = 1,000 and 2,000 s/mm2) are used.

Preprocessing

Preprocessing software	Details can be found in Miller et al. (doi:10.1038/nn.4393) and Alfaro-Almagro et al. (https://doi.org/10.1016/j.neuroimage.2017.10.034).
Normalization	Details can be found in Miller et al. (doi:10.1038/nn.4393) and Alfaro-Almagro et al. (https://doi.org/10.1016/j.neuroimage.2017.10.034).
Normalization template	Details can be found in Miller et al. (doi:10.1038/nn.4393) and Alfaro-Almagro et al. (https://doi.org/10.1016/j.neuroimage.2017.10.034).
Noise and artifact removal	Details can be found in Miller et al. (doi:10.1038/nn.4393) and Alfaro-Almagro et al. (https://doi.org/10.1016/j.neuroimage.2017.10.034).
Volume censoring	Details can be found in Miller et al. (doi:10.1038/nn.4393) and Alfaro-Almagro et al. (https://doi.org/10.1016/j.neuroimage.2017.10.034).

Statistical modeling & inference

Model type and settings	Details can be found in Miller et al. (doi:10.1038/nn.4393) and Alfaro-Almagro et al. (https://doi.org/10.1016/j.neuroimage.2017.10.034).
Effect(s) tested	Details can be found in Miller et al. (doi:10.1038/nn.4393) and Alfaro-Almagro et al. (https://doi.org/10.1016/j.neuroimage.2017.10.034).

Specify type of analysis: ☐ Whole brain ☐ ROI-based ☒ Both

Anatomical location(s)	We used a multi-modal parcellation of human cerebral cortex developed in Glasser et al., 2016 (10.1038/nature18933).
Statistic type for inference (See Eklund et al. 2016)	Inference was not carried out when generating imaging phenotypes.
Correction	Inference was not carried out when generating imaging phenotypes.

Models & analysis

n/a	Involved in the study
☐	☒ Functional and/or effective connectivity
☒	☐ Graph analysis
☒	☐ Multivariate modeling or predictive analysis

Functional and/or effective connectivity	Functional connectivity
